# Survival landscape of different tumor regression grades and pathologic complete response in rectal cancer after neoadjuvant therapy based on reconstructed individual patient data

**DOI:** 10.1186/s12885-021-08922-1

**Published:** 2021-11-13

**Authors:** Jia-yi Li, Xuan-zhang Huang, Peng Gao, Yong-xi Song, Xiao-wan Chen, Xing-er Lv, Yv Fu, Qiong Xiao, Shi-yv Ye, Zhen-ning Wang

**Affiliations:** Department of Surgical Oncology and General Surgery, Key Laboratory of Precision Diagnosis and Treatment of Gastrointestinal Tumors, Ministry of Education, The First Affiliated Hospital of China Medical University, 155 North Nanjing Street, Heping District, Shenyang City, 110001 China

**Keywords:** Rectal cancer, Neoadjuvant therapy, Pathological complete response, Tumor regression grade, Survival

## Abstract

**Background:**

Neoadjuvant therapy can lead to different tumor regression grades (TRG) in rectal cancer after neoadjuvant therapy. The purposes of this study are to investigate the relationships among TRG, pathologic complete response (pCR) and long-term survival, on the basis of reconstructed individual patient data (IPD).

**Methods:**

The PubMed, Embase, Ovid and Cochrane CENTRAL databases were searched. The primary endpoint was to evaluate the survival landscape of different TRGs after neoadjuvant therapy and the secondary endpoint was to evaluate the associations between pCR and survival. IPD were reconstructed with Kaplan–Meier curves.

**Results:**

The 10-year overall survival (OS) and 5-year disease-free survival (DFS) were clearly higher in the pCR group than in the non-pCR (npCR) group (80.5% vs. 48.3, 90.1% vs. 69.8%). Furthermore, the OS and DFS increased with improvement in tumor regression after neoadjuvant therapy. According to the IPD, the pCR group had longer OS (HR = 0.240, 95% CI = 0.177–0.325, *p* < 0.001) and DFS (HR = 0.274, 95% CI = 0.205–0.367, p < 0.001) than the npCR group. Better tumor regression was associated with better survival outcomes (*p* < 0.005). Direct calculation of published HR values yielded similar results.

**Conclusions:**

Our results indicate a positive relationship between better tumor regressions and improved survival benefits among the npCR group and patients with rectal cancer achieving pCR had much longer OS and DFS than patients achieving npCR, presenting a survival landscape of different TRGs and pCR in rectal cancer after neoadjuvant therapy.

**Supplementary Information:**

The online version contains supplementary material available at 10.1186/s12885-021-08922-1.

## Background

Colorectal cancer is the third most commonly diagnosed cancer and the second leading cause of cancer death [1]. In the NCCN guidelines, neoadjuvant treatment followed by surgery is the standard treatment for patients with locally advanced rectal cancer [2]. However, the CAO/ARO/AIO-94 trial has found that neoadjuvant treatment improves only the local control rate and disease-free survival (DFS) but not overall survival (OS) [3]. Our previous study indicated that neoadjuvant treatment is sufficient for local tumor control but not for prolonging OS [4]. Unfortunately, these previous studies evaluated only the long-term survival outcomes between patients receiving and not receiving neoadjuvant treatment and ignored the differences in tumor response to neoadjuvant treatment.

Neoadjuvant treatment can lead to differences in tumor regression in patients with rectal cancer because different tumor regression grades (TRGs) depend on tumor sensitivity to neoadjuvant treatment [5], and pathological complete response (pCR) can occur in well-responding patients. Compared with long-term survival outcomes, pCR is easier to obtain directly and accurately, and thus is usually used as a predictor of survival outcomes in clinical trials of neoadjuvant treatment. Exploration of the relationship between pCR and long-term survival outcomes is important for clinical practice. Some studies have demonstrated that achieving pCR after neoadjuvant treatment is associated with greatly improved clinical outcomes in patients with rectal cancer [6–8]. However, a pooled analysis assessing the role pCR as an alternative endpoint for survival in rectal cancer treated with neoadjuvant therapy has demonstrated that pCR is not an alternative endpoint for 5-year survival [9]. Some studies have reported that pCR does not improve survival. Pucciarelli et al. have shown that pCR following preoperative chemoradiation therapy for middle to lower rectal cancer is not a predictive factor for better outcomes, and Tseng et al. have also indicated that pCR and tumor downstaging do not improve outcomes [10, 11]. Therefore, exploration of whether pCR is a suitable predictor for neoadjuvant treatment is urgently needed.

Additionally, the number of patients with rectal cancer achieving pCR after neoadjuvant treatment is limited (14–20%) [11–13]; instead, most patients with rectal cancer achieve non-pCR (npCR) after neoadjuvant treatment. The prognostic value of tumor regression in determining survival benefits in patients with rectal cancer after neoadjuvant treatment is a matter of debate. In the CAO/ARO/AIO-04 trial, a relationship between better TRG after neoadjuvant treatment and more favorable clinical outcomes in patients with rectal cancer has been observed [12]. However, another randomized controlled trial found that TRG has no prognostic value for DFS in patients with rectal cancer after neoadjuvant treatment [14]. Therefore, further exploration of the influence of different TRGs on survival benefits in patients with rectal cancer after neoadjuvant treatment is needed.

The primary endpoint of this meta-analysis was to investigate the survival rates and outcomes in patients with rectal cancer achieving different TRGs after neoadjuvant treatment by using extracted individual patient data (IPD), and the secondary endpoint was to further assess the relationship between pCR and long-term survival benefits.

## Methods

This meta-analysis was conducted in accordance Preferred Reporting Items for Systematic Reviews and Meta-Analyses (PRISMA) Guideline and was registered in PROSPERO (ID: CRD42020215007).

### Search strategy

We searched PubMed, Embase, Ovid and Cochrane CENTRAL databases for relevant studies published through September 2020. The MeSH/main keywords of the search strategy included the following terms: “neoadjuvant chemotherapy,” “neoadjuvant radiotherapy,” “neoadjuvant chemoradiotherapy,” “neoadjuvant treatment,” “neoadjuvant therapy,” “preoperative chemotherapy,” “preoperative radiotherapy,” “preoperative chemoradiotherapy,” “preoperative treatment,” “preoperative therapy,” “pre-operative chemotherapy,” “pre-operative radiotherapy,” “pre-operative chemoradiotherapy,” “pre-operative treatment,” “pre-operative therapy,” “rectal cancer,” “colorectal cancer,” “tumor regression grade,” “tumor response,” “tumor respond” “pathological complete response,” “pathological complete respond,” “pathological complete remission,” “TRG,” “pCR,” “survival,” “prognosis,” “outcome,” “recurrence,” “relapse,” “disease-free survival” and “survival rate.” If several studies reported on the same population, only the most recent study was included. Reference lists of relevant studies were also reviewed to identify additional studies.

### Eligibility criteria

The inclusion criteria for studies were as follows: (i) patients were diagnosed with rectal cancer; (ii) all patients underwent preoperative neoadjuvant therapy followed by surgery; (iii) studies contained two or more pathological TRGs; and (iv) studies reported the outcomes of OS or DFS with Kaplan-Meier curves and numbers at risk. The exclusion criteria for studies were as follows: (i) patients were diagnosed with other types of cancer; (ii) patients did not receive preoperative neoadjuvant treatment or surgery; (iii) studies reported one TRG or fewer; (iv) studies lacked eligible outcomes; (v) studies did not report Kaplan-Meier curves or numbers at risk; and (vi) studies were reviews, case reports or meta-analyses.

### Definition of tumor regression after neoadjuvant treatment and clinical endpoints

The TRG was first proposed in 1993 [15] and has been generally used as a marker of tumor response to neoadjuvant therapy in clinical practice and many clinical studies, and is recommended by NCCN guidelines. TRG is determined by the amount of tumor and fibrotic tissue in the tumor embedding area after neoadjuvant therapy, which is easy to evaluate. Several TRG classification systems have been introduced in studies, and the percentage of fibrotic tissue and residual tumor used to define the grades of tumor regression varies among TRG classification systems. Thus, because variations exist among studies, comprehensive comparison of survival among different grades of tumor regression after neoadjuvant therapy is difficult. Here, we evaluated and regrouped patients according to similar grades of tumor regression of each TRG criterion to present the results more succinctly and intuitively.

The pCR was defined as ypT0N0M0 or no residual tumor cells. Patients who had not achieved pCR were placed in the npCR group. For the npCR group, near pCR was defined as Dworak-TRG 3, AJCC-TRG 1 or 50% ≤ tumor regression < 100%. Good regression was defined as Dworak-TRG 3–4, AJCC-TRG 0–1 or 50% ≤ tumor regression ≤100% (pCR group + near pCR group). Poor regression was defined as Dworak-TRG 0–2, AJCC-TRG 2–3 and tumor regression < 50%. Moderate regression was defined as Dworak-TRG 2–3 and AJCC-TRG 1–2. Major regression was defined as Dworak-TRG 2–4 and AJCC-TRG 0–2 (pCR group + moderate regression group). Minor regression was defined as Dworak-TRG 0–1 or AJCC-TRG 3. The npCR group was defined as (near pCR group + poor regression group) or (moderate regression group + minor regression group). The details are shown in Supplementary Fig. 1.

DFS was defined as the time between randomization and either local or distant relapse or death. OS was defined as the time from randomization to death from any cause.

### Data extraction

All eligible studies were reviewed and assessed by two authors, and data extraction was completed independently. All disagreements were resolved by comprehensive discussion. We recorded the author, publication year, country, number of patients, study design, age, definition of pCR, different groups of tumor regression, follow-up duration, TNM stage, neoadjuvant treatment regimen, adjuvant treatment regimen and study outcomes, including OS and DFS. Moreover, the number of events and corresponding numbers at risk at different times in the Kaplan-Meier curves of the included studies were obtained with Digitizer software, and the number of events and corresponding numbers at risk were used for reconstructing IPD according to the method of Guyot et al. [16]. IPD is time-to-event data including follow-up time and time-dependent event of each patient, which could be used for survival analysis.

### Statistical analysis

The primary clinical goals of this meta-analysis were to evaluate the survival rates (i.e., OS and DFS) at different years and the corresponding hazard ratio (HR) according to different TRGs after neoadjuvant therapy. The secondary clinical endpoints were the clinical associations between pCR and survival after neoadjuvant therapy, including survival rates (OS and DFS) at different years and the corresponding HR between the pCR and npCR groups. Overall analyses were conducted by including all relevant studies. To assess the potential effects of relevant factors including study design type, neoadjuvant regimen and follow-up duration (5-year time frame) on survival outcomes of patients with rectal cancer after neoadjuvant therapy, we performed subgroup analyses based on these factors.

To visually evaluate the survival rate by pooling time-to-event outcomes, we reconstructed IPD from the Kaplan-Meier curves of the included studies according to the method of Guyot et al. [16], which is a close approximation to the original IPD. This analytical method converts digitized Kaplan-Meier curves into IPD by finding numerical solutions to the inverted Kaplan-Meier equations, a procedure requiring the number of events and corresponding numbers at risk. Thus, we first used Digitizer software to obtain the number of events and corresponding numbers at risk from the Kaplan-Meier curve and then used the MASS, splines and survival packages in R software to calculate the IPD for further survival analysis. To determine the specific survival rates in different years and the median survival times, we conducted Kaplan-Meier analyses to reconstruct and pool Kaplan-Meier survival curves by using IPD, and we obtained the annual survival rate of OS and DFS for patients in each group. Additionally, corresponding IPD-derived HR values for survival outcomes (i.e., OS and DFS) were evaluated from IPD by using the Cox regression model, considering that HR was a comparative value and could represent the probability of survival benefits in the intervention group compared with the control group in the follow-up period. To confirm the reliability of the IPD results, we also directly calculated an estimated HR by pooling published HR values, and the HR or 95% confidence interval (CI) was obtained through published data by using the methods reported by Tierney et al. [17] if they were not directly reported.

The Cochran Q test and I^2^ statistic were used to evaluate the heterogeneity among the studies [18]. The heterogeneity was considered statistically significant when I^2^ > 50% or *p* < 0.1. A random effects model was used if the heterogeneity was significant; otherwise, a fixed-effects model was used. We constructed funnel plots with Begg’s and Egger’s tests to assess the publication bias [19, 20].

*P* values less than 0.05 were considered statistically significant. All analyses were performed with SPSS (version 16.0), R Studio (version 6.3) and Stata (version 12.0).

## Results

### Study selection

**Fig. 1:**
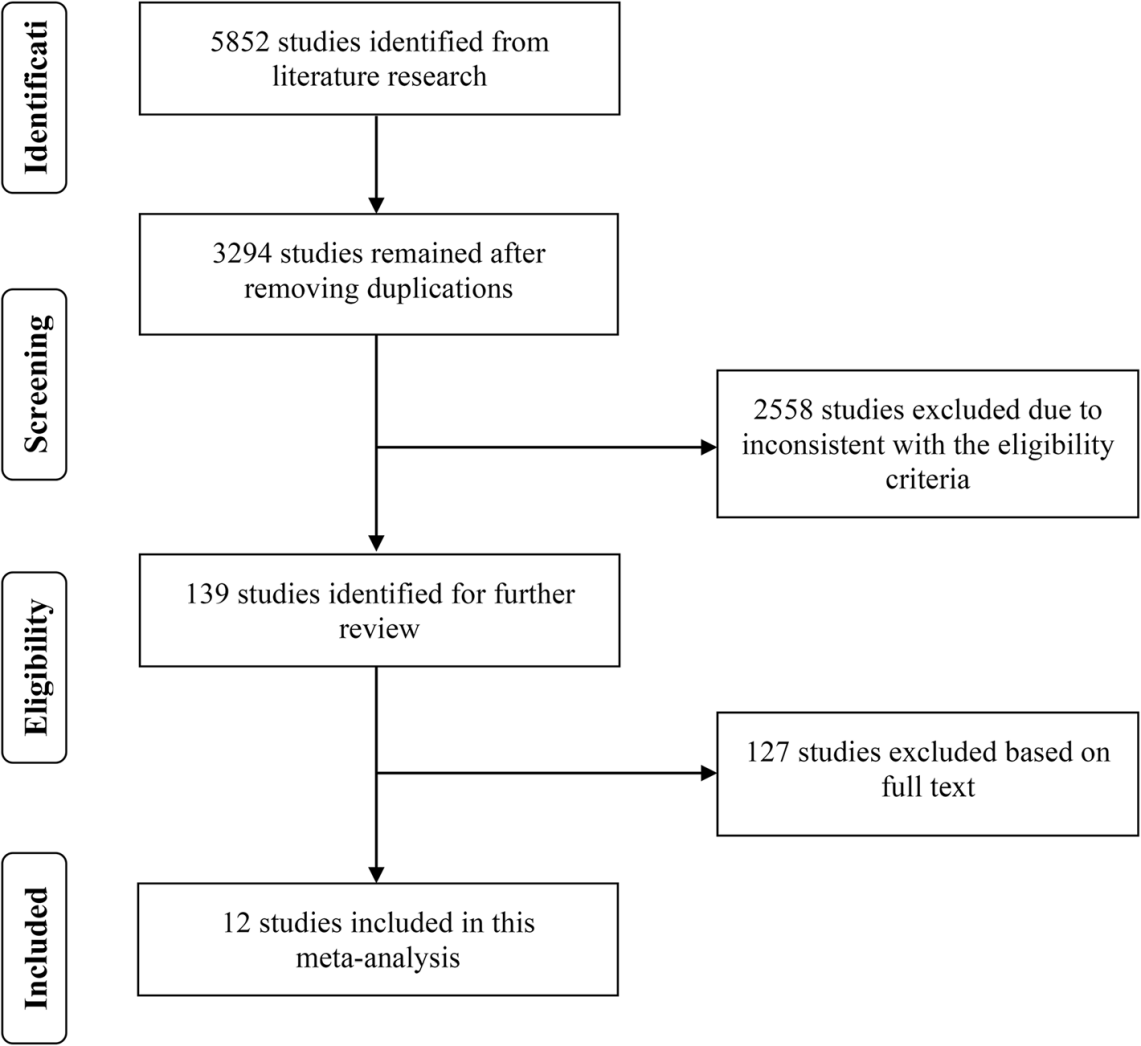
Literature search and study selection

A total of 5852 relevant studies were identified from PubMed, Embase, Ovid and Cochrane CENTRAL. A total of 3294 articles remained after elimination of duplicates. After review of the titles and abstracts, 139 articles were retained according to the eligibility criteria. Finally, 12 articles were included in this meta-analysis after review of the full text [6–8, 12, 14, 21–27]. The included studies comprised four randomized controlled trials, two prospective studies, two post hoc studies of prospective data and four retrospective studies. The detailed flowchart is shown in Fig. 1.

### Characteristics of the included studies

The 12 included studies were published between 2010 and 2019, and examined a total of 5931 patients. Among the included studies, five provided a pCR group and npCR group; one provided a pCR group, npCR group, near pCR group, good regression group and poor regression group; two provided a pCR group, npCR group, moderate regression group, major regression group and minor regression group; and four provided a pCR group, npCR group, near pCR group, good regression group, moderate regression group, major regression group, poor regression group and minor regression group. The follow-up duration was from 3.6 months to 15.8 years. The baseline characteristics of the included studies are shown in Table 1.

**Table 1:** The baseline characteristics of included studies

Study	Country&year	Study design	NO. of patients (M/F)	Age mean ± SD/median (range)	Definition of pCR	Different groups of tumor regression	Follow-up median (range)	TNM stage (0/I/II/III)	Neoadjuvant treatment regimen	Outcome
Erlandsson	Sweden 2019	RCT	697 (416/281)	NR	ypT0N0M0	①②③④⑤	5.7y (IQR, 4.9–14.3y)	yp:42/213/215/201	RT	OS, DFS
Marco	USA 2018	Prospective study	211 (124/87)	NR	No residual tumor cells	①②	59 m (9–125 m)	yp:65/58/41/47	RT + FU/ RT + FU + mFOLFOX6	OS, DFS
Song	Korea 2018	Retrospective study	331 (229/102)	≤61y: *n* = 161 >61y: *n* = 170	No residual tumor cells	①②③④⑤⑥⑦⑧	65.0 m (8.4–159.3 m)	yp:45/94/80/112	RT + CAP/ RT + FU + LV/ RT + cetuximab + irinotecan + capecitabine	OS, DFS
Karagkounis	USA 2018	Post hoc study	305 (224/81)	57.5y(25.9–85.9y)	No residual tumor cells	①②③④⑤⑥⑦⑧	4.9y (range 0.3–15.8y)	yp:−/123/182/−	RT + FU/ RT + CAP	OS
Kuan	China 2017	Retrospective study	1914 (1300/614)	59.97 ± 12.10y: *n* = 1655 59.59 ± 12.36: *n* = 259	ypT0N0M0	①②	37.0 m	c:−/523/1391/−	RT + FU/ LV, tegafur or capecitabine	OS
Fokas	German 2017	RCT	1179 (838/341)	NR	No residual tumor cells	①②⑥⑦⑧	50 m (38–61 m)	NR	RT + FU/ RT + FU + OX	OS, DFS
De Felice	Italy 2016	Prospective study	100 (67/33)	64y (38-76y)	No residual tumor cells	①②	NR	NR	RT + OX + FU	DFS
Zhang	China 2015	Retrospective study	295 (203/92)	<55y: *n* = 153 ≥55y: *n* = 142	ypT0N0M0	①②③④⑤⑥⑦⑧	36 m (5–120 m)	yp:77/53/97/68	RT + XELOX/ RT + FOLFOX/ RT + Xeloda	OS, DFS
Fokas	German 2014	RCT	391 (283/108)	≤61y: *n* = 205 >61y: *n* = 186	No residual tumor cells	①②⑥⑦⑧	132 m (90–184 m)	NR	RT + FU	DFS
de Campos-Lobato	Brazil 2011	Post hoc study	238 (174/64)	57y (49–67y)	ypT0N0M0	①②	55 m (IQR, 36–77 m)	NR	RT + 5-FU/ RT + CAP	OS, DFS
Belluco	Italy 2011	Retrospective study	139 (93/46)	62y	No residual tumor cells	①②	55.4 m	NR	RT + 5-FU + LV/ RT + 5-FU + gefitinib/ RT + CAP/ RT + CAP + OX/ RT + raltitrexed	DFS
Bujko	Poland 2010	RCT	131 (88/43)	TRG 0: 58y (39–72y) TRG 1: 62y (44–70y) TRG 2: 59y (41–72y) TRG 3: 59y (34–73y)	No residual tumor cells	①②③④⑤	4y	NR	RT + FU + LV	DFS

*M/F* male/female; *pCR* pathological complete response; *RCT* randomized control trial; *m* month; *y* year; *TRG* tumor regression grade; *IQR* interquartile range; ①: pCR group; ②: non-pCR group; ③: near pCR group; ④: good regression group; ⑤: poor regression group; ⑥: major regression group; ⑦: moderate regression group; ⑧:minor regression group; *c* clinical stage; *yp* pathologic stage after receiving neoadjuvant chemotherapy; *RT* radiotherapy; *FU* fluorouracil; *mFOLFOX6* fluorouracil + leucovorin + oxaliplatin; *FOLFOX* fluorouracil + leucovorin + oxaliplatin; *LV* leucovorin; *CAP* capecitabine; *OX* oxaliplatin; *XELOX* capecitabine + oxaliplatin; *OS* overall survival; *DFS* disease-free survival; *NR* not reported

### OS and tumor regressions to neoadjuvant treatment

**Fig. 2:**
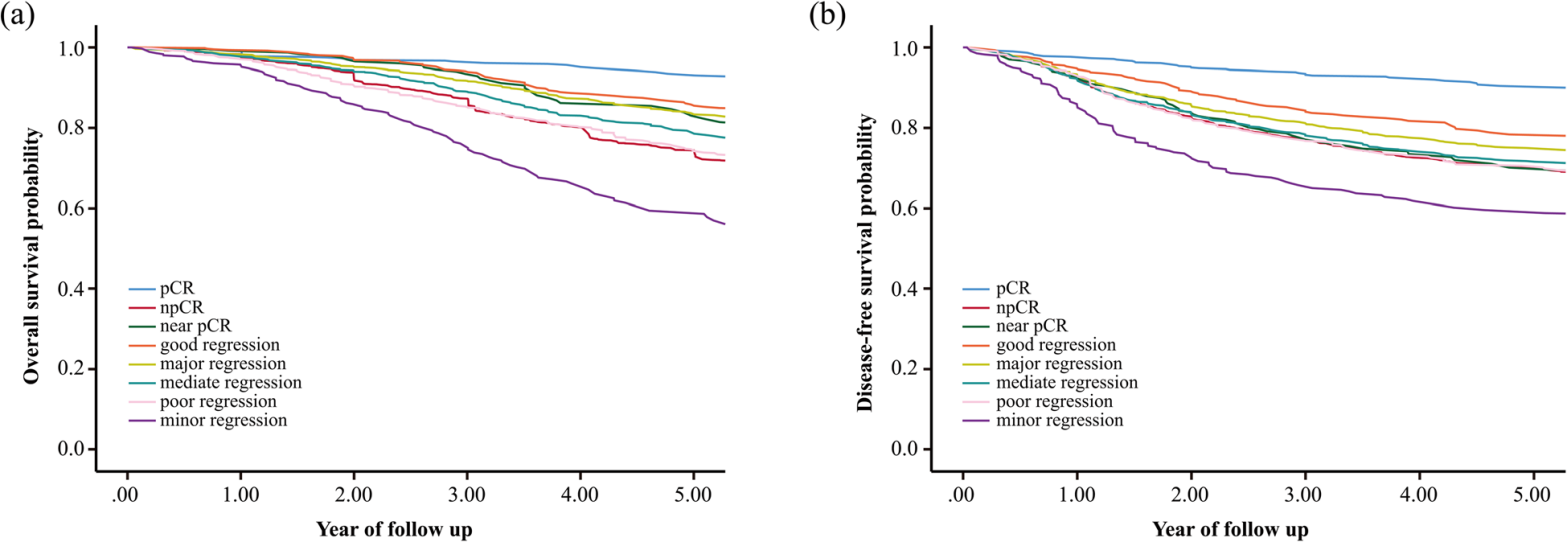
The pooled Kaplan–Meier curves for 5-year survival outcomes (**a**) Overall survival; (**b**) Disease-free survival

The pooled Kaplan-Meier curves for OS indicated no clear difference in 1-year OS (> 95%) between different TRG groups (Fig. 2a and Supplementary Table 1). According to the Kaplan-Meier curves, a higher 5-year OS was observed in the pCR group than the other groups, and better tumor regression after neoadjuvant treatment contributed to longer 5-year OS (Supplementary Table 1). Moreover, to evaluate the long-term survival benefits from neoadjuvant treatment, we chose 10-year OS as an endpoint. The 10-year OS of the pCR group, good regression group, near pCR group, major regression group, moderate regression group, npCR group, poor regression group and minor regression group was 80.5, 61.1, 53.6, 55.9, 50.9, 48.3, 46.1 and 20.9%, respectively. A significantly longer OS was observed in the pCR group than the other groups, and this trend was more pronounced with increasing follow-up. In addition, we assessed the median survival time in each group. Patients in the npCR group had a median survival time of 9.458 years (95% CI: 8.843–10.074), whereas the median survival time in the moderate regression group, poor regression group and minor regression group was 10.244 years (95% CI: 8.679–11.808), 9.192 years (95% CI: 8.546–9.837) and 6.485 years (95% CI: 5.273–7.698) (Supplementary Table 1), respectively.

The HR values calculated with IPD indicated that better tumor regression was clearly associated with longer OS. Patients who achieved pCR showed longer OS than patients in the npCR group (HR = 0.240, 95% CI = 0.177–0.325), near pCR group (HR = 0.418, 95% CI = 0.271–0.646), moderate regression group (HR = 0.301, 95% CI = 0.218–0.415), poor regression group (HR = 0.235, 95% CI = 0.172–0.323) and minor regression group (HR = 0.132, 95% CI = 0.094–0.187). Compared with poor regression group, longer OS was observed in the near pCR group (HR = 0.561, 95% CI = 0.405–0.776) and good regression group (HR = 0.470, 95% CI = 0.375–0.589). In addition, the near pCR group (HR = 0.317, 95% CI = 0.222–0.453), moderate regression group (HR = 0.439, 95% CI = 0.356–0.541) and major regression group (HR = 0.333, 95% CI = 0.269–0.412) had longer OS values than the minor regression group. The detailed HRs for IPD are shown in Table 2. Given that the HR was calculated from the Cox proportional hazards model, which may be affected by follow-up duration, and certain 5-year survival outcomes have been generally used as main clinical endpoint in clinical studies, we performed subgroup analysis by limiting the follow-up duration to 5 years. This process provided an explicit time frame of survival outcomes, and the results similarly indicated that better tumor regression contributed to longer OS (Supplementary Table 2).

**Table 2:** Survival outcomes of comparison between groups

		IPD				Direct calculation				
		HR	LCI	UCI	p for HR	HR	LCI	UCI	p for HR	Heterogeneity (P, I^2^)
**OS**	pCR vs. npCR	0.240	0.177	0.325	< 0.001	0.391	0.322	0.475	< 0.001	0.953, 0.0%
	pCR vs. Near	0.418	0.271	0.646	< 0.001	0.406	0.220	0.749	0.004	0.813, 0.0%
	pCR vs. Moderate	0.301	0.218	0.415	< 0.001	0.400	0.310	0.517	< 0.001	0.837, 0.0%
	pCR vs. Poor	0.235	0.172	0.323	< 0.001	0.364	0.257	0.516	< 0.001	0.777, 0.0%
	pCR vs. Minor	0.132	0.094	0.187	< 0.001	0.249	0.173	0.359	< 0.001	0.360, 67.0%
	Near vs. Poor	0.561	0.405	0.776	< 0.001	0.654	0.501	0.853	0.002	0.116, 49.3%
	Good vs. Poor	0.470	0.375	0.589	< 0.001	0.546	0.430	0.695	< 0.001	0.646, 0.0%
	Near vs. Minor	0.317	0.222	0.453	< 0.001	0.375	0.254	0.553	< 0.001	0.164, 44.6%
	Moderate vs. Minor	0.439	0.356	0.541	< 0.001	0.412	0.321	0.529	< 0.001	0.312, 15.9%
	Major vs. Minor	0.333	0.269	0.412	< 0.001	0.316	0.187	0.534	< 0.001	0.039, 64.0%
**DFS**	pCR vs. npCR	0.260	0.195	0.347	< 0.001	0.421	0.350	0.506	< 0.001	0.890, 0.0%
	pCR vs. Near	0.277	0.190	0.402	< 0.001	0.403	0.251	0.648	< 0.001	0.990, 0.0%
	pCR vs. Moderate	0.282	0.209	0.381	< 0.001	0.462	0.363	0.588	< 0.001	0.814, 0.0%
	pCR vs. Poor	0.261	0.193	0.353	< 0.001	0.362	0.260	0.505	< 0.001	0.476, 0.0%
	pCR vs. Minor	0.175	0.126	0.243	< 0.001	0.251	0.183	0.344	< 0.001	0.517, 0.0%
	Near vs. Poor	0.941	0.716	1.237	0.663	0.660	0.490	0.889	0.006	0.205, 34.5%
	Good vs. Poor	0.634	0.496	0.811	< 0.001	0.541	0.415	0.706	< 0.001	0.453, 0.0%
	Near vs. Minor	0.629	0.464	0.852	0.003	0.415	0.179	0.958	0.039	0.149, 52.0%
	Moderate vs. Minor	0.618	0.504	0.757	< 0.001	0.583	0.462	0.736	< 0.001	0.894, 0.0%
	Major vs. Minor	0.535	0.437	0.654	< 0.001	0.460	0.359	0.588	< 0.001	0.756, 0.0%

*IPD* individual patient data; *HR* hazard ratio; *LCI* lower 95% confidence interval; *UCI* upper 95% confidence interval; *I*^*2*^ degree of heterogeneity; *OS* overall survival; *DFS* disease-free survival; *pCR* pathological complete response group; *npCR* non-pCR group; *Near* near pCR group; *Moderate* moderate regression group; *Poor* poor regression group; *Minor* minor regression group; *Good* good regression group; *Major* major regression group

**Fig. 3:**
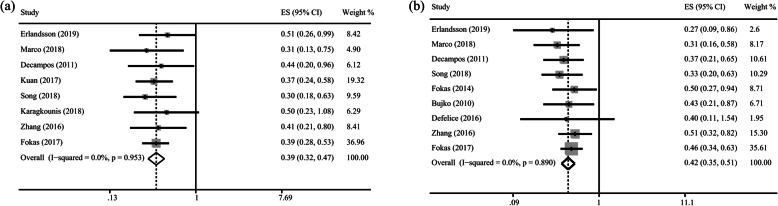
Forest plot based on survival outcomes (**a**) Overall survival in pCR group versus npCR group; (**b**) Disease-free survival in pCR group versus npCR group

The estimated HR, calculated directly by using published HR values, showed similar results, thus verifying the accuracy and reliability of the results obtained from IPD. According to the HR results calculated through direct combination in Supplementary Fig. 2 and Table 2, patients in pCR group had longer OS than patients in the npCR group (Fig. 3a), near pCR group, moderate regression group, poor regression group and minor regression group (*p* < 0.005). The OS was significantly longer in the near pCR group (HR = 0.654, 95% CI = 0.501–0.853) and good regression group (HR = 0.546, 95% CI = 0.430–0.695) than the poor regression group. In addition, longer OS was achieved in the near pCR group (HR = 0.375, 95% CI = 0.254–0.553), moderate regression group (HR = 0.412, 95% CI = 0.321–0.529) and major regression group (HR = 0.316, 95% CI = 0.187–0.534) than the minor regression group.

As relevant factors affecting the pathological response to neoadjuvant therapy, study design types and neoadjuvant regimens should be accounted for in the analysis. Thus, we performed further subgroup analyses based on a prospective study design and neoadjuvant regimen by using IPD-based data and directly published HR data. The results were similar to those of the overall analyses, thus indicating that better tumor regression was associated with favorable OS (Supplementary Table 3 and Supplementary Table 4).

### DFS and tumor regressions to neoadjuvant treatment

The pooled Kaplan-Meier curves for DFS are shown in Fig. 2b, and the annual DFS rates are shown in Supplementary Table 1. The 1-year DFS for the pCR group, good regression group, near pCR group, major regression group, moderate regression group, npCR group, poor regression group and minor regression group was 97.7, 94.8, 92.7, 93.4, 92.3, 92.3, 92.0 and 86.0%, respectively. The pCR group had longer 5-year DFS, than the other npCR groups, and the TRG was generally associated with 5-year DFS (Supplementary Table 1).

According to the extracted IPD, we calculated the HR values to compare the survival benefits in terms of DFS between groups. DFS was significantly higher in the pCR group than the npCR group (HR = 0.260, 95% CI = 0.195–0.347), near pCR group (HR = 0.277, 95% CI = 0.190–0.402), moderate regression group (HR = 0.282, 95% CI = 0.209–0.381), poor regression group (HR = 0.261, 95% CI = 0.193–0.353) and minor regression group (HR = 0.175, 95% CI = 0.126–0.243). Patients in the good regression group had longer DFS than patients in the poor regression group (HR = 0.634, 95% CI = 0.496–0.811). Longer DFS was observed in the near pCR group (HR = 0.629, 95% CI = 0.464–0.852), moderate regression group (HR = 0.618, 95% CI = 0.504–0.757) and major regression group (HR = 0.535, 95% CI = 0.437–0.654) than the minor regression group. The detailed HRs for IPD are shown in Table 2. Additionally, we performed subgroup analysis of the DFS with a 5-year follow-up duration, and the results also indicated that better tumor regression contributed to longer OS (Supplementary Table 2).

To confirm the reliability of the IPD, we directly calculated estimated HR by pooling published HR values. According to the HR results calculated through direct combination in Supplementary Fig. 3 and Table 2, patients with rectal cancer who achieved pCR had a longer DFS than patients in the npCR group (Fig. 3b), near pCR group, moderate regression group, poor regression group and minor regression group, thus confirming the accuracy and reliability of IPD results. Moreover, patients in the near pCR group (HR = 0.660, 95% CI = 0.490–0.889) and good regression group (HR = 0.541, 95% CI = 0.415–0.706) achieved longer DFS than patients in the poor regression group. In addition, DFS was longer in the near pCR group (HR = 0.415, 95% CI = 0.179–0.958), moderate regression group (HR = 0.583, 95% CI = 0.462–0.736) and major regression group (HR = 0.460, 95% CI = 0.359–0.588) than the minor regression group.

The results of subgroup analyses based on a prospective study design and neoadjuvant regimen (Supplementary Table 3 and Supplementary Table 4) confirmed the association between survival benefits and different TRG groups.

## Discussion

In this meta-analysis, the OS and DFS in the pCR group were significantly higher than those in the npCR group, and group with better tumor regression after neoadjuvant treatment had higher survival rates. Additionally, we used IPD-derived HR to evaluate the survival benefits in different groups by using reconstructed IPD, and the results indicated that better tumor regression led to survival benefits, and a finding was also demonstrated in subgroup analyses based on study types and neoadjuvant regimens.

We found that the survival benefit in the pCR group was more significant and obvious than that in the npCR group after longer follow-up time. Patients with rectal cancer who achieve pCR have less local recurrence and distant failure [28], possibly because of an absence of residual tumor cells in patients with pCR, thereby decreasing the risk of tumor recurrence and metastasis, and leading to better survival rates. Moreover, the varied tumor response to neoadjuvant therapy suggests a complex relationship between tumor biology and tumor response. Several studies have found that pCR is associated with favorable tumor factors, such as good histology, lower grade, T and N classification, and negative CEA [29–31], and many genes or molecular pathways have been found to regulate chemoradiotherapy sensitivity [32, 33]. Therefore, patients in the pCR group might have better tumor biological characteristics than those in the npCR group, who were less sensitive to chemoradiotherapy, owing to certain genetic or molecular expression and pathways [33]. Thus, better survival benefits were easier to achieve through clinical treatment. Furthermore, the 10-year OS of the pCR group (80.5%) was significantly higher than that of the other groups and notably was four times that of the minor regression group (20.9%). Therefore, pCR can be used as a reliable predictor for assessing long-term survival outcomes.

Although pCR is the expected response after neoadjuvant treatment, the prognosis of npCR is also worthy of attention. The effects of neoadjuvant treatment varied among patients with rectal cancer with npCR. Cancer can be graded according to differences in tumor regression, corresponding to different prognoses. According to our results, patients with rectal cancer who had better regression after neoadjuvant treatment clearly achieved better survival outcomes, a finding consistent with results reported by Fokas and colleagues [12]. This observation may have been because the classification of tumor regression was based on a decrease in tumor volume, and therefore patients with better tumor regression had better tumor characteristics and were more sensitive to neoadjuvant treatment. They were also less likely to relapse or undergo metastasis, and were more likely to show benefits in survival outcomes. Our results indicated that physicians should consider the tumor response and TRG after neoadjuvant treatment during the treatment of rectal cancer. However, the evaluation criteria for TRG are inconsistent across studies; therefore, more studies are needed to standardize the criteria in the future.

The strength of this study lies in the reconstruction of IPD from the Kaplan-Meier curves of the included studies to systematically and accurately evaluate pooled survival rates (i.e., OS and DFS) at different years, median survival times and corresponding HR values according to different TRGs after neoadjuvant therapy. We utilized the reconstructed IPD to draw more comprehensive, powerful and reliable results and conclusions. The included studies used several TRG classification systems as criteria to evaluate the tumor response to neoadjuvant therapy; thus, the definitions of tumor response grades vary among the studies. Therefore, we divided the npCR patients into several groups according to the different TRG criteria of tumor response to neoadjuvant therapy and further compared their survival benefits among different groups. In addition, this study included all eligible clinical studies containing RCT, prospective and retrospective clinical trials on the associations between tumor response to neoadjuvant therapy and long-term survival benefits. To reduce bias, we performed in-depth subgroup analyses based on the study design type and neoadjuvant regimen to enhance the reliability of our results and conclusions.

Recently, total neoadjuvant therapy (TNT) has become an alternative therapeutic strategy involving administration of all chemotherapy and chemoradiotherapy before surgery [34]. Standard neoadjuvant chemoradiotherapy and TNT are two completely different sequencing therapy strategies, and neoadjuvant strategies may affect pathologic tumor responses. Indeed, several studies have compared the pathologic tumor response between the standard neoadjuvant chemoradiotherapy and TNT, and shown that TNT results in more favorable pathologic tumor responses [35–37]. Thus, simple meta-analysis of the two therapy strategies may result in substantial heterogeneity. Additionally, several studies reported that the time between neoadjuvant chemoradiotherapy and surgery is associated with the rate of pCR [38, 39] and thus may be an important factor affecting the results on the association of TGR and survival in patients with rectal cancer after neoadjuvant therapy. However, the included studies did not report information about the time between neoadjuvant chemoradiotherapy and surgery, and thus subgroup analysis to examine this relationship could not be performed, owing to insufficient data. Thus, future large-scale, prospective clinical studies are needed to assess the effects of TNT and time between neoadjuvant chemoradiotherapy and surgery on the associations between tumor response and survival.

A watch-and-wait approach has been proposed as an alternative therapy choice to avoid rectal cancer surgery for patients with complete response after neoadjuvant therapy, given the absence of significant differences in non-regrowth recurrence and survival benefits between the watch-and-wait approach and surgery [40, 41]. However, the proportion of the patients with a complete response who underwent a watch-and-wait approach was small, because most patients choose a watch-and-wait approach mainly because of refusal or high risk of surgery; surgery is still the main standard therapy strategy after neoadjuvant therapy. To reduce the bias caused by different treatment regimens after neoadjuvant therapy and enhance the homogeneity of the study considering the influence of different treatment strategies on survival, patients who underwent the watch-and-wait approach were excluded from this analysis, and the exclusion did not affect our present results and conclusions. Future well-designed prospective clinical studies are required to definitively evaluate the safety of the watch-and-wait approach for rectal cancer patients with complete response after neoadjuvant therapy.

The present study has several limitations. First, some potentially confounding bias could not be eliminated, owing to the retrospective nature of the study. Second, the IPD used in the analysis were obtained by calculation and may therefore differ from the real IPD. Third, the use of adjuvant chemotherapy is a factor that may affect long-term survival in patients receiving neoadjuvant therapy. However, there were no uniform therapy criteria for postoperative adjuvant chemotherapy among the included studies and only some of the included patients received adjuvant chemotherapy in our study. Nine included studies administrated adjuvant chemotherapy in some of the patients, one study administrated adjuvant chemotherapy in all patients, one study did not administrate adjuvant chemotherapy, and the remaining study did not report the detailed adjuvant regimens. However, we were unable to extract available data on adjuvant chemotherapy, and therefore we were unable to perform in-depth subgroup analysis to examine this aspect, owing to insufficient data. Future large-scale, well-designed, prospective studies are needed to investigate the effects of adjuvant chemotherapy regimens on the association between tumor response and survival. Moreover, lymph node invasion may have affected the prognosis of patients with rectal cancer, but the data were not provided for further analysis. Finally, the heterogeneity among studies could not be eliminated. In the future, more high-quality studies are needed to investigate and address these problems.

## Conclusions

Patients with rectal cancer achieving pCR after neoadjuvant treatment and surgery had longer OS and DFS than those achieving npCR; thus, pCR can be considered a reliable predictor. Moreover, better tumor regression after neoadjuvant treatment was significantly associated with better clinical outcomes in patients with rectal cancer. The results provide new evidence for clinical practice to accurately identify patients who would benefit from neoadjuvant treatment, thus selecting further individual treatment strategies based on tumor regression after neoadjuvant treatment.

## Supplementary Information

**Additional file 1: Fig. S1.**

**Additional file 2: Fig. S2.**

**Additional file 3: Fig. S3.**

**Additional file 4: Table S1.**

**Additional file 5: Table S2.**

**Additional file 6: Table S3.**

**Additional file 7: Table S4.**

## Data Availability

The data that support the findings of this study are available from the corresponding author upon reasonable request.

## Abbreviations

DFS: Disease-free survival
OS: Overall survival
TRG: Tumor regression grade
pCR: pathological complete response
npCR: non-pCR
IPD: Individual patient data
HR: Hazard ratio
CI: Confidence interval
TNT: Total neoadjuvant therapy
