# Supplementary figures and images for: Survival landscape of different tumor regression grades and pathologic complete response in rectal cancer after neoadjuvant therapy based on reconstructed individual patient data
Source: BMC Cancer. 2021 Nov 13;21:1214. doi: 10.1186/s12885-021-08922-1 (PMC8590217; doi:10.1186/s12885-021-08922-1)

| <b>Tumor regression %</b> | 100 | $50 \leq \text{TR} < 100$ | <50 |     |
|---------------------------|-----|---------------------------|-----|-----|
| <b>Dworak-TRG</b>         | 4   | 3                         | 2   | 1+0 |
| <b>AJCC-TRG</b>           | 0   | 1                         | 2   | 3   |

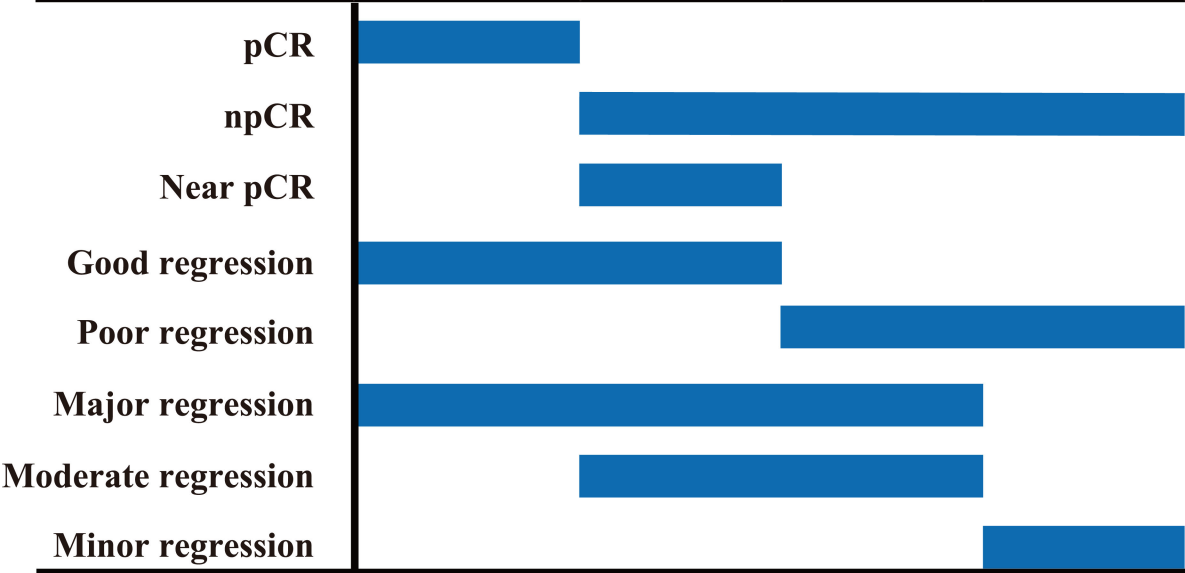

Supplement: Supplementary file 1 — Additional file 1: Fig. S1. [file 12885_2021_8922_MOESM1_ESM.pdf]

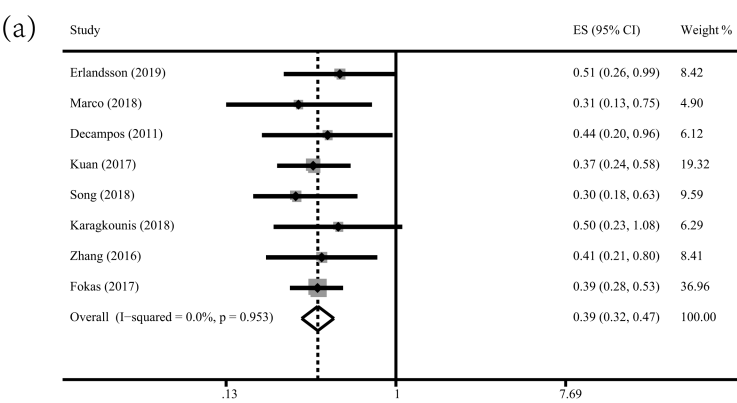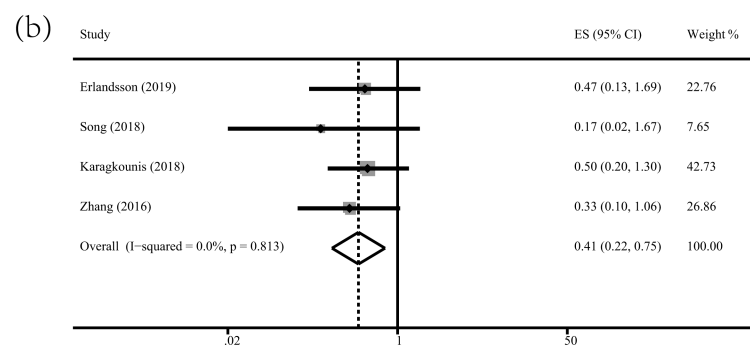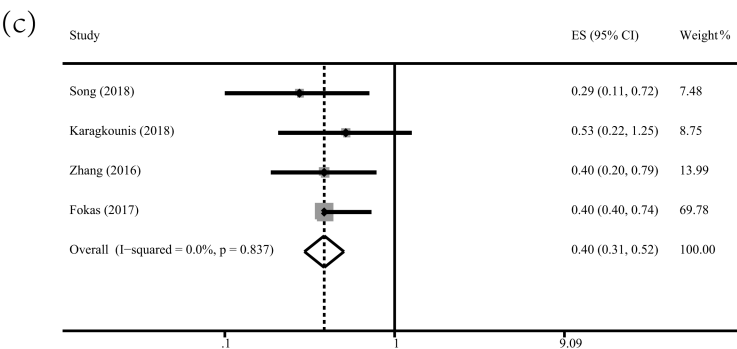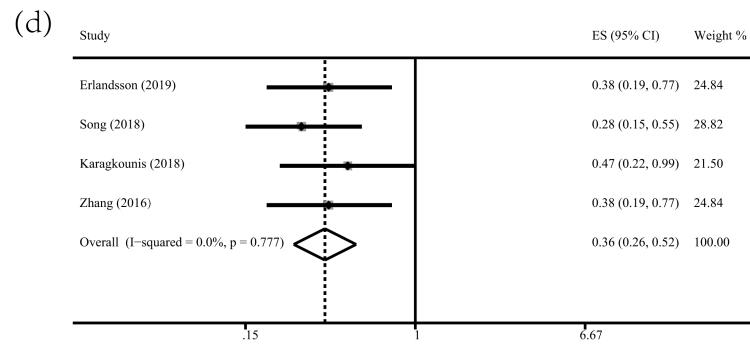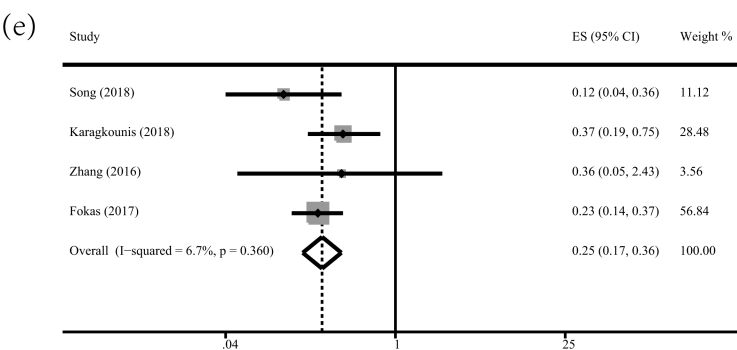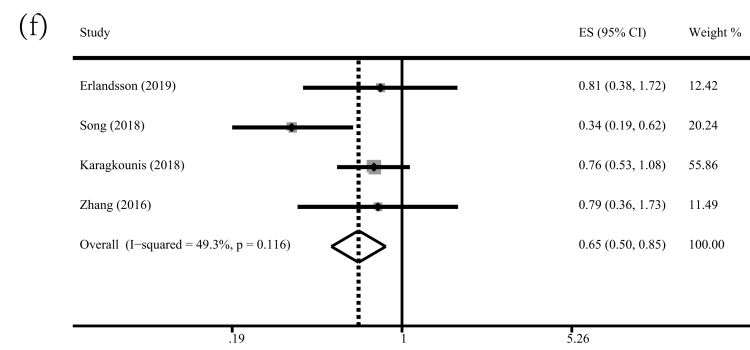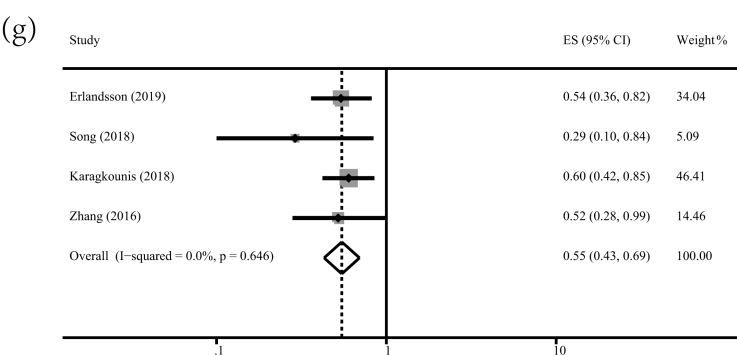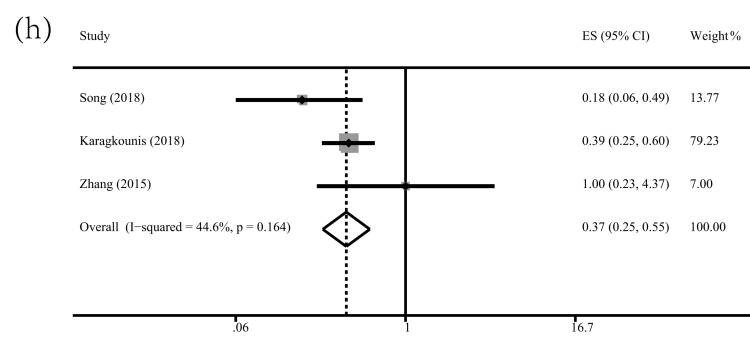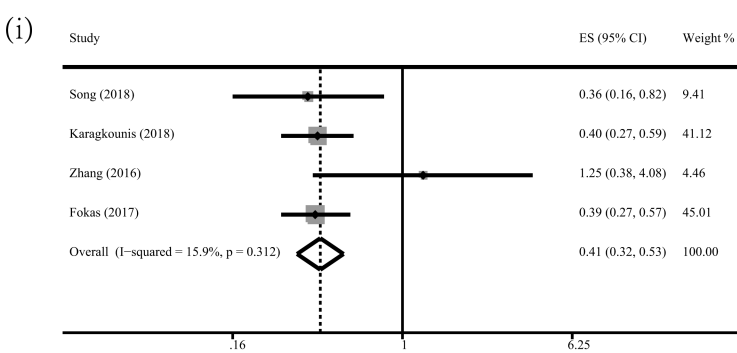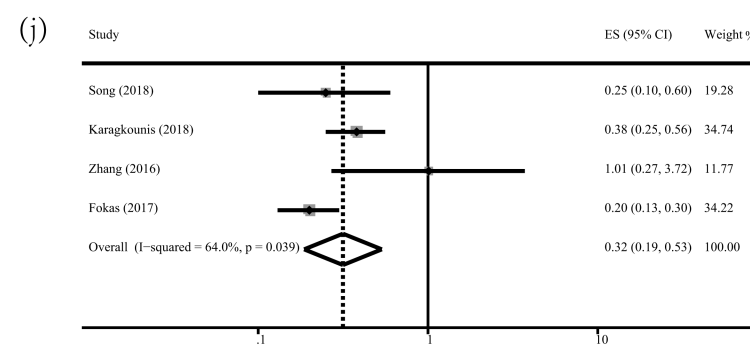

Supplement: Supplementary file 2 — Additional file 2: Fig. S2. [file 12885_2021_8922_MOESM2_ESM.pdf]

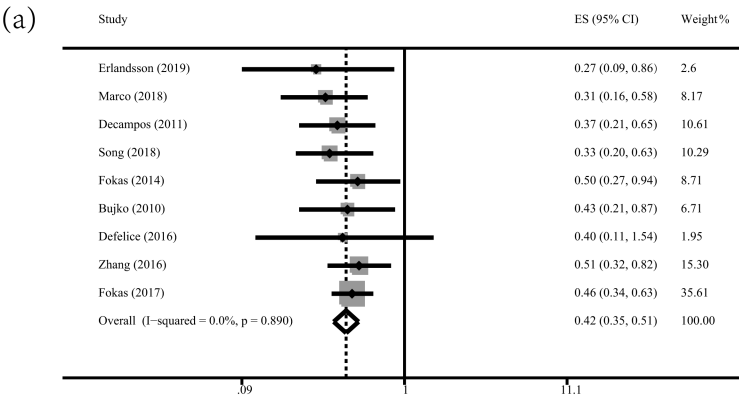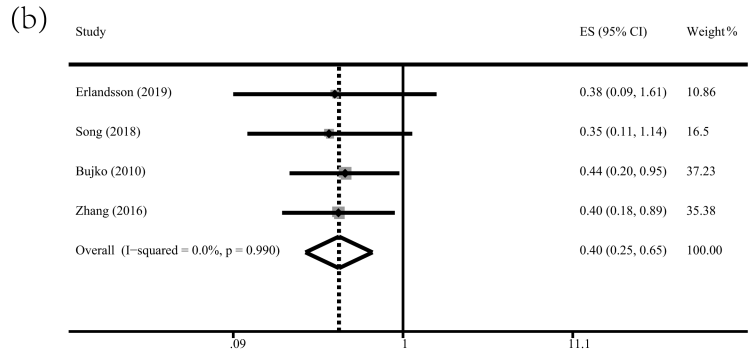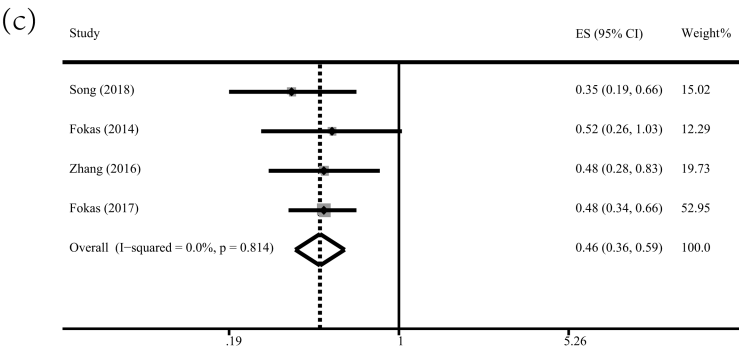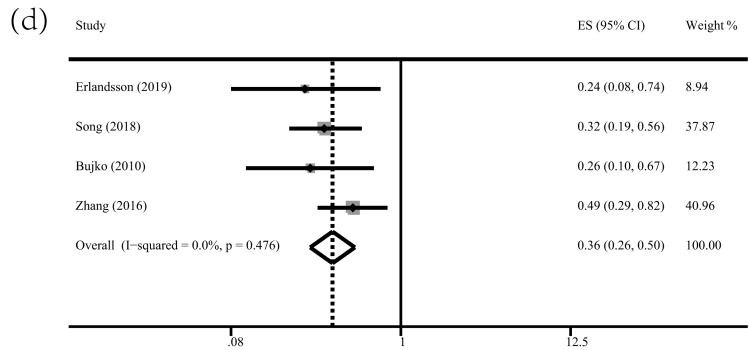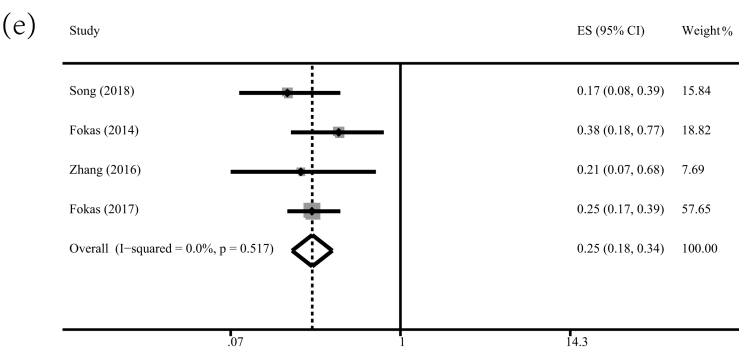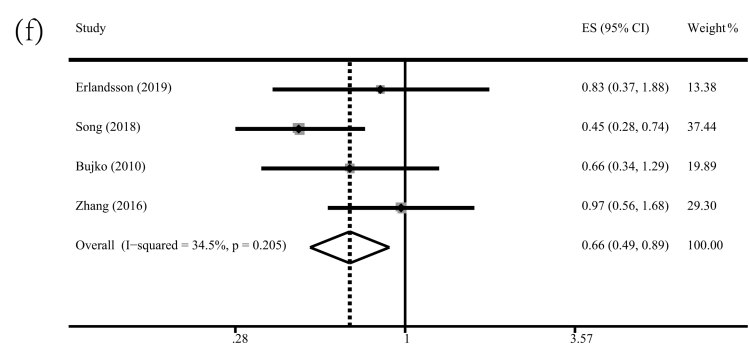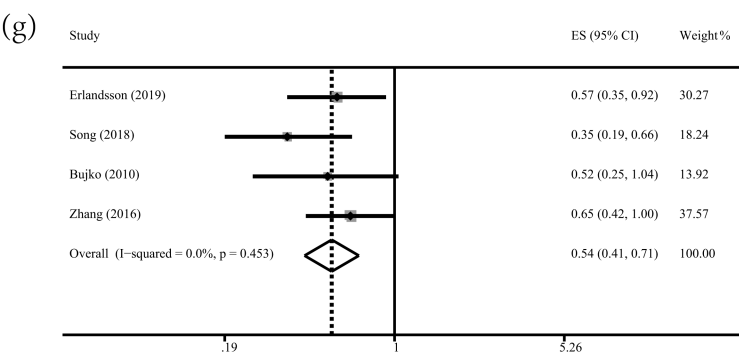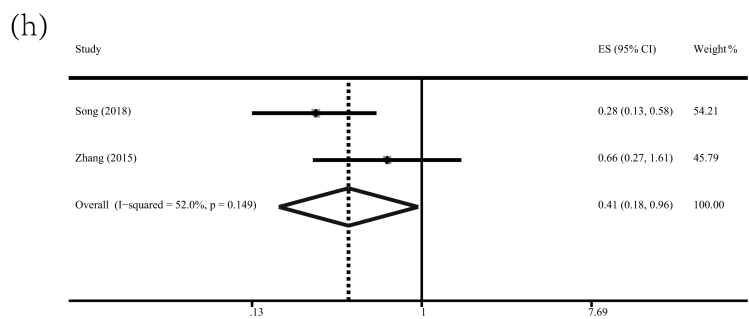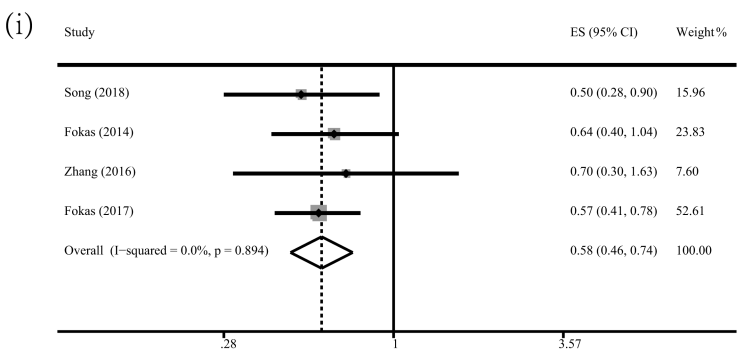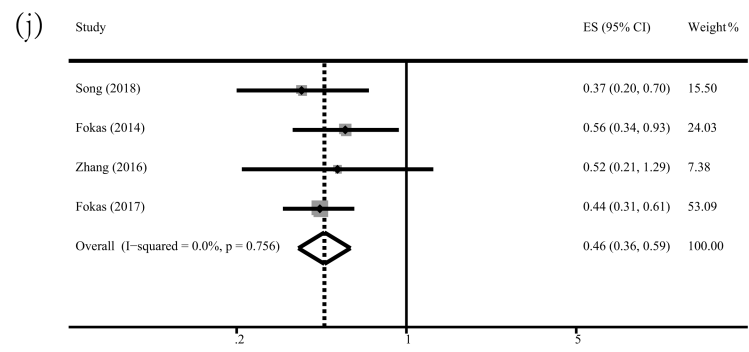

Supplement: Supplementary file 3 — Additional file 3: Fig. S3. [file 12885_2021_8922_MOESM3_ESM.pdf]
