# Supplementary material for: Survival landscape of different tumor regression grades and pathologic complete response in rectal cancer after neoadjuvant therapy based on reconstructed individual patient data
Source: BMC Cancer. 2021 Nov 13;21:1214. doi: 10.1186/s12885-021-08922-1 (PMC8590217; doi:10.1186/s12885-021-08922-1)
Supplement: Supplementary file 4 — Additional file 4: Table S1. [file 12885_2021_8922_MOESM4_ESM.pdf]

Supplementary Table1. Annual survival rate of each group

	Group	Survival rate %										Median survival time (range, year)		
		1-year	2-year	3-year	4-year	5-year	6-year	7-year	8-year	9-year	10-year			
**OS**	pCR	99.5	97.3	96.5	95.1	93.5	90.0	90.0	90.0	80.5	80.5		/	
	Good regression	99.4	96.9	94.1	88.7	85.8	82.9	81.3	77.3	61.1	61.1		/	
	Near pCR	99.2	96.5	93.4	86.2	83.1	80.4	79.3	71.7	53.6	53.6		/	
	Major regression	98.3	95.1	91.8	87.4	83.7	79.5	76.2	71.2	60.5	55.9		/	
	Moderate regression	97.9	94.2	89.0	83.1	78.9	74.0	70.7	63.0	53.0	50.9	10.244	8.679	11.808
	npCR	97.6	92.0	87.3	80.3	74.4	68.3	63.6	57.9	52.3	48.3	9.458	8.843	10.074
	Poor regression	97.1	90.8	85.3	80.2	74.3	68.1	61.9	54.8	50.8	46.1	9.192	8.546	9.837
	Minor regression	95.7	85.9	75.4	66.2	59.4	51.8	47.3	39.0	33.5	20.9	6.485	5.273	7.698
**DFS**	pCR	97.5	94.6	92.7	92.0	90.1	/	/	/	/	/		/	
	Good regression	94.8	89.2	84.0	81.8	78.4	/	/	/	/	/		/	
	Near pCR	92.7	83.9	78.6	74.8	71.7	/	/	/	/	/		/	
	Major regression	93.4	85.7	81.2	77.5	75.0	/	/	/	/	/		/	
	Moderate regression	92.3	83.7	78.1	74.1	71.6	/	/	/	/	/		/	
	npCR	92.2	82.4	76.6	72.4	69.8	/	/	/	/	/		/	
	Poor regression	92.0	81.9	76.4	72.3	69.5	/	/	/	/	/		/	
	Minor regression	86.0	72.7	65.4	61.8	58.9	/	/	/	/	/		/	

pCR: pathological complete response; npCR: non-pCR; “/”: there is no relevant data
