# Supplementary material for: Survival landscape of different tumor regression grades and pathologic complete response in rectal cancer after neoadjuvant therapy based on reconstructed individual patient data
Source: BMC Cancer. 2021 Nov 13;21:1214. doi: 10.1186/s12885-021-08922-1 (PMC8590217; doi:10.1186/s12885-021-08922-1)
Supplement: Supplementary file 5 — Additional file 5: Table S2. [file 12885_2021_8922_MOESM5_ESM.pdf]

Supplementary Table 2. 5-year survival outcomes of comparison between groups

		IPD			
		HR	LCI	UCI	p for HR
**OS**	pCR vs. npCR	0.233	0.166	0.327	<0.001
	pCR vs. Near	0.406	0.249	0.661	<0.001
	pCR vs. Moderate	0.294	0.206	0.419	<0.001
	pCR vs. Poor	0.235	0.165	0.333	<0.001
	pCR vs. Minor	0.124	0.085	0.182	<0.001
	Near vs. Poor	0.578	0.397	0.843	0.004
	Good vs. Poor	0.496	0.382	0.643	<0.001
	Near vs. Minor	0.308	0.206	0.460	<0.001
	Moderate vs. Minor	0.424	0.339	0.530	<0.001
	Major vs. Minor	0.317	0.253	0.398	<0.001
**DFS**	pCR vs. npCR	0.269	0.201	0.360	<0.001
	pCR vs. Near	0.276	0.189	0.404	<0.001
	pCR vs. Moderate	0.288	0.213	0.389	<0.001
	pCR vs. Poor	0.272	0.200	0.369	<0.001
	pCR vs. Minor	0.175	0.125	0.244	<0.001
	Near vs. Poor	0.978	0.742	1.289	0.874
	Good vs. Poor	0.665	0.519	0.853	0.001
	Near vs. Minor	0.627	0.461	0.852	0.003
	Moderate vs. Minor	0.607	0.494	0.747	<0.001
	Major vs. Minor	0.523	0.427	0.641	<0.001

IPD: individual patient data; HR: hazard ratio; LCI: lower 95% confidence interval; UCI: upper 95% confidence interval; I^2: degree of heterogeneity; OS: overall survival; DFS: disease-free survival; pCR: pathological complete response group; npCR: non-pCR group; Near: near pCR group; Moderate: moderate regression group; Poor: poor regression group; Minor: minor regression group; Good: good regression group; Major: major regression group
