# Supplementary material for: Survival landscape of different tumor regression grades and pathologic complete response in rectal cancer after neoadjuvant therapy based on reconstructed individual patient data
Source: BMC Cancer. 2021 Nov 13;21:1214. doi: 10.1186/s12885-021-08922-1 (PMC8590217; doi:10.1186/s12885-021-08922-1)
Supplement: Supplementary file 6 — Additional file 6: Table S3. [file 12885_2021_8922_MOESM6_ESM.pdf]

Supplementary Table 3. Survival outcomes of comparison between groups based on prospective study design

		IPD				Direct calculation				
		HR	LCI	UCI	p for HR	HR	LCI	UCI	p for HR	Heterogeneity (P, I^2)
**OS**	pCR vs. npCR	0.200	0.130	0.309	<0.001	0.412	0.322	0.526	<0.001	0.882, 0.0%
	pCR vs. Near	0.273	0.155	0.482	<0.001	0.489	0.230	1.042	0.064	0.939, 0.0%
	pCR vs. Moderate	0.243	0.155	0.381	<0.001	0.413	0.309	0.552	<0.001	0.549, 0.0%
	pCR vs. Poor	0.202	0.130	0.315	<0.001	0.419	0.251	0.700	0.001	0.685, 0.0%
	pCR vs. Minor	0.103	0.064	0.164	<0.001	0.270	0.181	0.401	<0.001	0.268, 18.5%
	Good vs. Poor	0.576	0.414	0.801	0.001	0.574	0.439	0.750	<0.001	0.703, 0.0%
	Near vs. Minor	0.369	0.246	0.554	<0.001	0.390	0.250	0.600	/	/
	Moderate vs. Minor	0.423	0.336	0.533	<0.001	0.395	0.301	0.517	<0.001	0.927, 0.0%
	Major vs. Minor	0.322	0.255	0.407	<0.001	0.276	0.147	0.518	<0.001	0.030, 78.7%
**DFS**	pCR vs. npCR	0.270	0.193	0.377	<0.001	0.419	0.338	0.519	<0.001	0.892, 0.0%
	pCR vs. Near	0.205	0.130	0.324	<0.001	0.426	0.214	0.845	0.015	0.861, 0.0%
	pCR vs. Moderate	0.294	0.207	0.418	<0.001	0.487	0.361	0.657	<0.001	0.837, 0.0%
	pCR vs. Poor	0.303	0.212	0.434	<0.001	0.251	0.122	0.518	<0.001	0.915, 0.0%
	pCR vs. Minor	0.182	0.124	0.267	<0.001	0.277	0.193	0.397	<0.001	0.327, 0.0%
	Good vs. Poor	1.001	0.719	1.393	0.994	0.554	0.371	0.826	0.004	0.834, 0.0%
	Near vs. Minor	0.885	0.608	1.289	0.525	/	/	/	/	/
	Moderate vs. Minor	0.619	0.489	0.783	<0.001	0.591	0.453	0.772	<0.001	0.693, 0.0%
	Major vs. Minor	0.538	0.427	0.679	<0.001	0.474	0.358	0.628	<0.001	0.436, 0.0%

IPD: individual patient data; HR: hazard ratio; LCI: lower 95% confidence interval; UCI: upper 95% confidence interval; I^2: degree of heterogeneity; OS: overall survival; DFS: disease-free survival; pCR: pathological complete response group; npCR: non-pCR group; Near: near

pCR group; Moderate: moderate regression group; Poor: poor regression group; Minor: minor regression group; Good: good regression group; Major: major regression group; “/”: not applicable due to limited data
