# Supplementary material for: Survival landscape of different tumor regression grades and pathologic complete response in rectal cancer after neoadjuvant therapy based on reconstructed individual patient data
Source: BMC Cancer. 2021 Nov 13;21:1214. doi: 10.1186/s12885-021-08922-1 (PMC8590217; doi:10.1186/s12885-021-08922-1)
Supplement: Supplementary file 7 — Additional file 7: Table S4. [file 12885_2021_8922_MOESM7_ESM.pdf]

Supplementary Table 4. Survival outcomes of comparison between groups based on RT + 5-FU-based-monotherapy neoadjuvant therapy

		IPD				Direct calculation				
		HR	LCI	UCI	p for HR	HR	LCI	UCI	p for HR	Heterogeneity (P, I^2)
**OS**	pCR vs. npCR	0.244	0.172	0.345	<0.001	0.384	0.308	0.478	<0.001	0.879, 0.0%
	pCR vs. Near	0.500	0.295	0.848	0.010	0.424	0.179	1.005	0.051	0.379, 0.0%
	pCR vs. Moderate	0.336	0.233	0.484	<0.001	0.400	0.303	0.528	<0.001	0.653, 0.0%
	pCR vs. Poor	0.220	0.151	0.321	<0.001	0.349	0.214	0.571	<0.001	0.307, 4.2%
	pCR vs. Minor	0.141	0.096	0.206	<0.001	0.246	0.169	0.357	<0.001	0.215, 34.9%
	Near vs. Poor	0.469	0.311	0.705	0.004	0.527	0.240	1.156	0.110	0.022, 80.8%
	Good vs. Poor	0.444	0.334	0.589	<0.001	0.558	0.400	0.780	0.001	0.204, 38.1%
	Near vs. Minor	0.291	0.192	0.442	<0.001	0.348	0.232	0.521	<0.001	0.183, 43.6%
	Moderate vs. Minor	0.417	0.336	0.518	<0.001	0.391	0.303	0.506	<0.001	0.974, 0.0%
	Major vs. Minor	0.317	0.255	0.396	<0.001	0.272	0.171	0.432	<0.001	0.093, 57.8%
**DFS**	pCR vs. npCR	0.255	0.174	0.374	<0.001	0.417	0.331	0.526	<0.001	0.751, 0.0%
	pCR vs. Near	0.310	0.192	0.502	<0.001	0.410	0.214	0.784	0.007	0.750, 0.0%
	pCR vs. Moderate	0.575	0.451	0.733	<0.001	0.448	0.334	0.600	<0.001	0.380, 0.0%
	pCR vs. Poor	0.229	0.149	0.351	<0.001	0.304	0.190	0.487	<0.001	0.710, 0.0%
	pCR vs. Minor	0.176	0.115	0.269	<0.001	0.230	0.159	0.332	<0.001	0.398, 0.0%
	Near vs. Poor	0.736	0.507	1.069	0.107	0.514	0.347	0.761	0.001	0.363, 0.0%
	Good vs. Poor	0.531	0.371	0.760	0.001	0.415	0.260	0.664	<0.001	0.412, 0.0%
	Near vs. Minor	0.565	0.390	0.818	0.003	0.280	0.133	0.591	/	/
	Moderate vs. Minor	0.306	0.207	0.452	<0.001	0.553	0.417	0.733	<0.001	0.700, 0.0%
	Major vs. Minor	0.490	0.386	0.624	<0.001	0.423	0.314	0.570	<0.001	0.633, 0.0%

RT: radiotherapy; FU: fluorouracil; IPD: individual patient data; HR: hazard ratio; LCI: lower 95% confidence interval; UCI: upper 95% confidence interval; I^2: degree of heterogeneity; OS: overall survival; DFS: disease-free survival; pCR: pathological complete response group; npCR: non-pCR group; Near: near pCR group; Moderate: moderate regression group; Poor: poor regression group; Minor: minor regression group; Good: good regression group; Major: major regression group; “/”: not applicable due to limited data
